# Accuracy of a novel calibratable real-time continuous glucose monitoring device based on FreeStyle libre in- and out-of-hospital

**DOI:** 10.3389/fendo.2025.1466358

**Published:** 2025-04-22

**Authors:** Zhenghao Wu, Zhaoxiang Liu, Wenhui Zhao, Shaocheng Wang, Liangbiao Gu, Jianzhong Xiao

**Affiliations:** ^1^ Department of Endocrinology, Beijing Tsinghua Changgung Hospital, School of Clinical Medicine, Tsinghua Medicine, Tsinghua University, Beijing, China; ^2^ Department of Endocrinology, The Quzhou Affiliated Hospital of Wenzhou Medical University, Quzhou Peoples Hospital, Quzhou, Zhejiang, China

**Keywords:** rtCGM, continuous glucose monitoring, accuracy, FreeStyle libre, calibrate

## Abstract

**Objectives:**

Based on FreeStyle Libre, we designed QT AIR, an advanced real-time, calibrated Continuous Glucose Monitoring (CGM) device. This study aim to validate the consistency and clinical accuracy of the product by comparing the capillary blood glucose (CBG) with CGM data in both in-hospital and outpatient scenarios.

**Methods:**

Results of CGM devices were compared with random capillary glucose values from users in both in-hospital and outpatient settings. The accuracy of CGMs was assessed through consistency analysis, Bland-Altman analysis, calculation of MARD and MAD, Consensus Error Grids, as well as analysis using the Continuous Glucose Deviation Interval and Variability Analysis (CG-DIVA).

**Results:**

In outpatient setting, 1907 values from 138 users were analyzed. FreeStyle Libre data, QT AIR calibrated and uncalibrated data showed strong positive correlations with capillary blood glucose values. The MARD values for the FreeStyle Libre, uncalibrated QT AIR, and calibrated QT AIR groups were 18.33%, 20.63%, and 12.39%, respectively. Consensus Error Grid, reference values in Zone A: FreeStyle Libre: 69.75%, QT AIR uncalibrated: 67.80%, QT AIR calibrated: 87.62%. The Bland-Altman analysis results suggest that FreeStyle Libre exhibitsed a systematic underestimation of blood glucose levels, while QT AIR almost rectified the differences. In the in-Hospital setting, the MARD of QT AIR after calibration was reduced to 7.24%. The Consensus error grid analyses of the in-Hospital data revealed that 95% of the calibrated QT AIR values fell within Zone A, a significantly higher proportion than that of other two group. The CG-DIVA analysis of the calibrated QT AIR device showed a median bias of -0.49% and a between-sensor variability of 26.65%, both of which are significantly lower than the corresponding values observed for the FreeStyle Libre device.

**Conclusions:**

We successfully transformed a retrospective CGM system into a real-time monitoring device. The monitoring accuracy of the device could be improved by calibration.

## 1 Introduction

Diabetes mellitus is a chronic metabolic disorder requiring long-term management (1). Currently, the population of diabetes is continuously increasing world-wide (2). The control of blood glucose (BG) has been shown to significantly reduce the complications of diabetes (3). Effective glycemic management relies upon close blood glucose monitoring. Continuous glucose monitoring (CGM) is an effective method that enables multi-day tracking of patients’ blood glucose levels without frequent blood sampling (4). It helps in the detection of asymptomatic hypoglycemic (or hyperglycemic) events that might be ignored by a self-monitoring of peripheral blood glucose (SMBG) (5). Furthermore, it generates an ambulatory glucose profile for the patient. The time in range (TIR) of blood glucose, time above range (TAR), and time below range (TBR) provided by CGM has now become a recommended target for glycemic management according to multiple guidelines (6).

**Figure 1:**
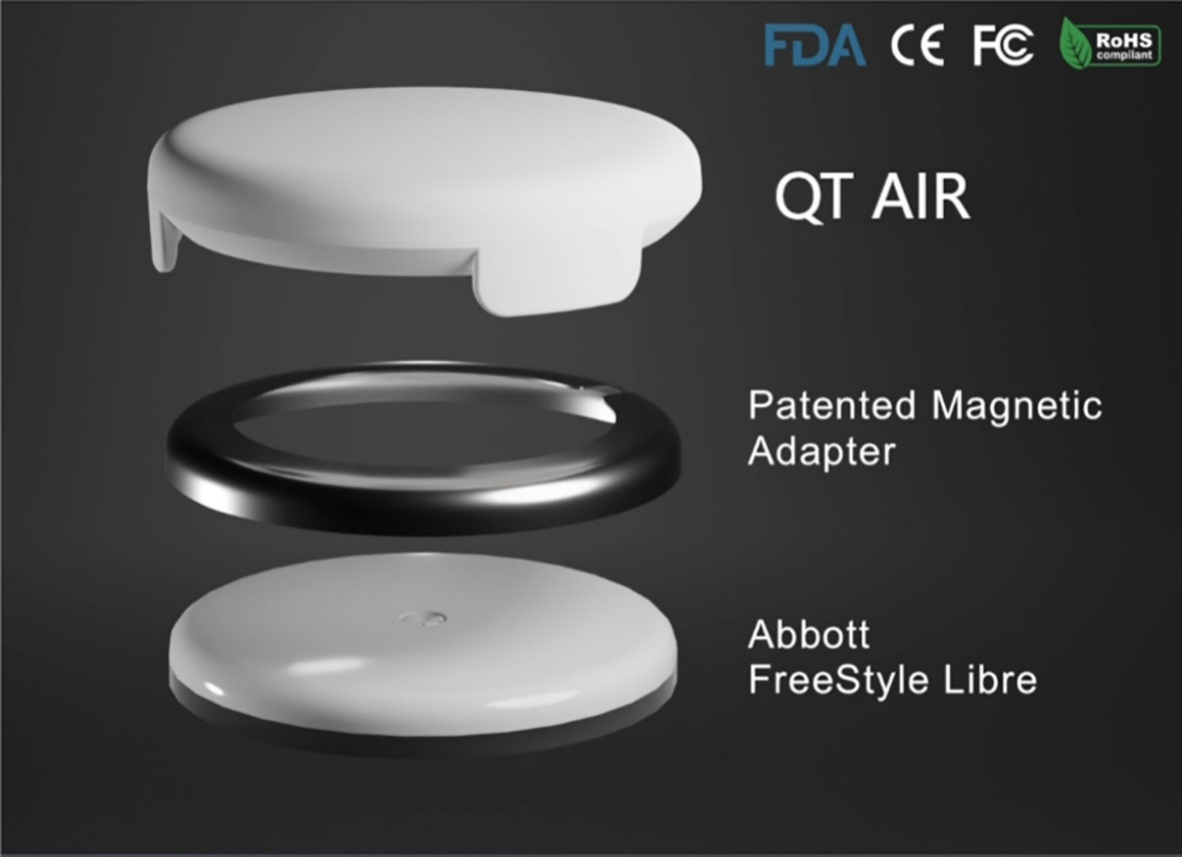
QT AIR 3D structure diagram.

FreeStyle^®^ Libre™ flash glucose monitoring (Abbott Diabetes Care, Alameda, CA) allows for convenient scanning to obtain instantaneous blood glucose readings and 14 days’ glucose profiles (7). Additionally, it records the blood glucose fluctuations of the preceding 8 hours at 15-minute intervals. FreeStyle Libre holds a significant market share in developing countries, including Mainland China. However, as an intermittent-scanning CGM, it still presents certain limitations:1) FreeStyle Libre requires an intermittent scan for data at least every 8 hours and is a retrospective glucose monitor that cannot provide real-time monitoring (8, 9). 2) Its clinical accuracy might be marginally insufficient, with glucose levels tending to be lower compared to readings from traditional blood glucose testings (10, 11). We have developed the “QT AIR” based on FreeStyle Libre to solve the problems of consistency of glucose with SMBG, the inability to calibrate and the inability to synchronize data, which can synchronize, calibrate and monitor patients’ blood glucose levels in real time. QT AIR is connected to FreeStyle Libre through a transfer hoop, capturing the electrical signal from FreeStyle Libre in real-time (**Figure 1**). Utilizing a proprietary intelligent algorithm, it promptly processes the data to generate glucose readings and subsequently transmits them to the cloud server. The QT APP available on both iOS and Android platforms, as well as the hospital’s QT AIR management PDA, can be paired with fingertip glucose meters. This enables for the synchronization and recording of users’ fingertip blood glucose measurements. Moreover, the fingertip glucose (FG) values are utilized to calibrate the monitoring readings of the QT AIR.

We aim to compare the consistency and clinical accuracy of clinical capillary blood glucose levels with the data obtained from Libre, QT AIR uncalibrated and QT AIR calibrated. This comparison will be conducted both in outpatient daily life settings and within the controlled environment of standard healthcare settings in hospitalized patients.

## 2 Methods

### 2.1 Patients

Adults with and without diabetes who wear QT AIR device and choose to consent to the use of the product to record blood glucose data were enrolled in this study.

The study encompasses two groups of participants:

a. Patients who wore QT AIR in outpatient settings (n = 138) from October 1, 2022, to March 31, 2023.

All device uers who electronically affirmed the QT APP End User License Agreement (EULA) were systematically enrolled in the randomized sampling framework.

b. Patients admitted to the Endocrinology Department at Beijing Tsinghua Chang Gung Hospital and wearing QT AIR (n = 38) from March, 2023, to March, 2024.

Eligible participants met the following criteria: (1) age ≥ 16 years with no gender restriction; (2) clinical indication for glycemic surveillance and willingness to maintain CGM device adherence for ≥7 consecutive days. Exclusion Criteria: (1) Required radiographic examinations (X-ray, MRI, or CT) or anticipated exposure to high-intensity radiation/electromagnetic fields during CGM application; (2) Presented with severe circulatory insufficiency; (3) Demonstrated documented hypersensitivity reactions to CGM sensor components; (4) Experienced acute metabolic complications including diabetic ketoacidosis (DKA); (5) Used pharmacotherapeutic agents with known interference potential on CGM or capillary blood glucose measurements.

### 2.2 Study design

Outpatient participants followed the instructions provided for wearing the Libre and the QT AIR. They downloaded the QT APP and activated and synchronized the devices through the app. Participants used their personal blood glucose meters, which were paired with the QT APP following authentication. This pairing process enables the automatic synchronization of self-measured fingertip blood glucose readings. Additionally, patients had the option to manually input their self-measured fingertip blood glucose values and corresponding test times into the QT APP. The reading in the stable blood glucose period (the glucose reading changes are lower than 0.05mmol/L·min) were utilized for calibrating the QT AIR data. During the wearing period, patients could have random fingertip blood glucose test. Before calibration, patients continued their daily routines and avoided strenuous exercise, eating, and injecting insulin for 3-4 hours, the blood glucose change rate should be less than 0.05 mmol/L·min. Concurrently, QT AIR uncalibrated data, QT AIR calibrated data, Libre readings, and fingertip blood glucose levels, were transmitted to and documented on a secure cloud-based server. In case of occurrences of hyperglycemia or hypoglycemia, the QT APP was able to alert the patients and provide feedback to healthcare providers, facilitating timely intervention to ensure comprehensive patient medical safety.

In the hospital setting, with the assistance of professional staff, the Libre and QT AIR were worn on the outer side of the upper arm. After initiating the Libre, patient and device information was inputted into the hospital’s internal glucose management platform. QT AIR device transmitted blood glucose readings to the hospital’s server at one-minute intervals. During the monitoring process, nursing staff applied the standard blood glucose meter (Accu-Chek^®^ Performa Connect) and utilized the same batch of blood glucose test strips for fingertip blood glucose measurements. All data will be systematically transmitted and recorded on the cloud server. If any hyperglycemic or hypoglycemic events occur, the hospital management platform will send immediate notifications to the doctor in charge. These notifications enable physicians to promptly intervene and make necessary adjustments to the treatment regimen.

Given that the FreeStyle Libre has a specified range of readings (2.2-27.8 mmol/L), data exceeding this range will be considered as output readings. The study will comprehensively compare the consistency and clinical accuracy of the data obtained from QT AIR uncalibrated, QT AIR calibrated, Libre readings, taking corresponding fingertip blood glucose values recorded at the matching time points as reference.

### 2.3 Statistical analysis

**Figure 2:**
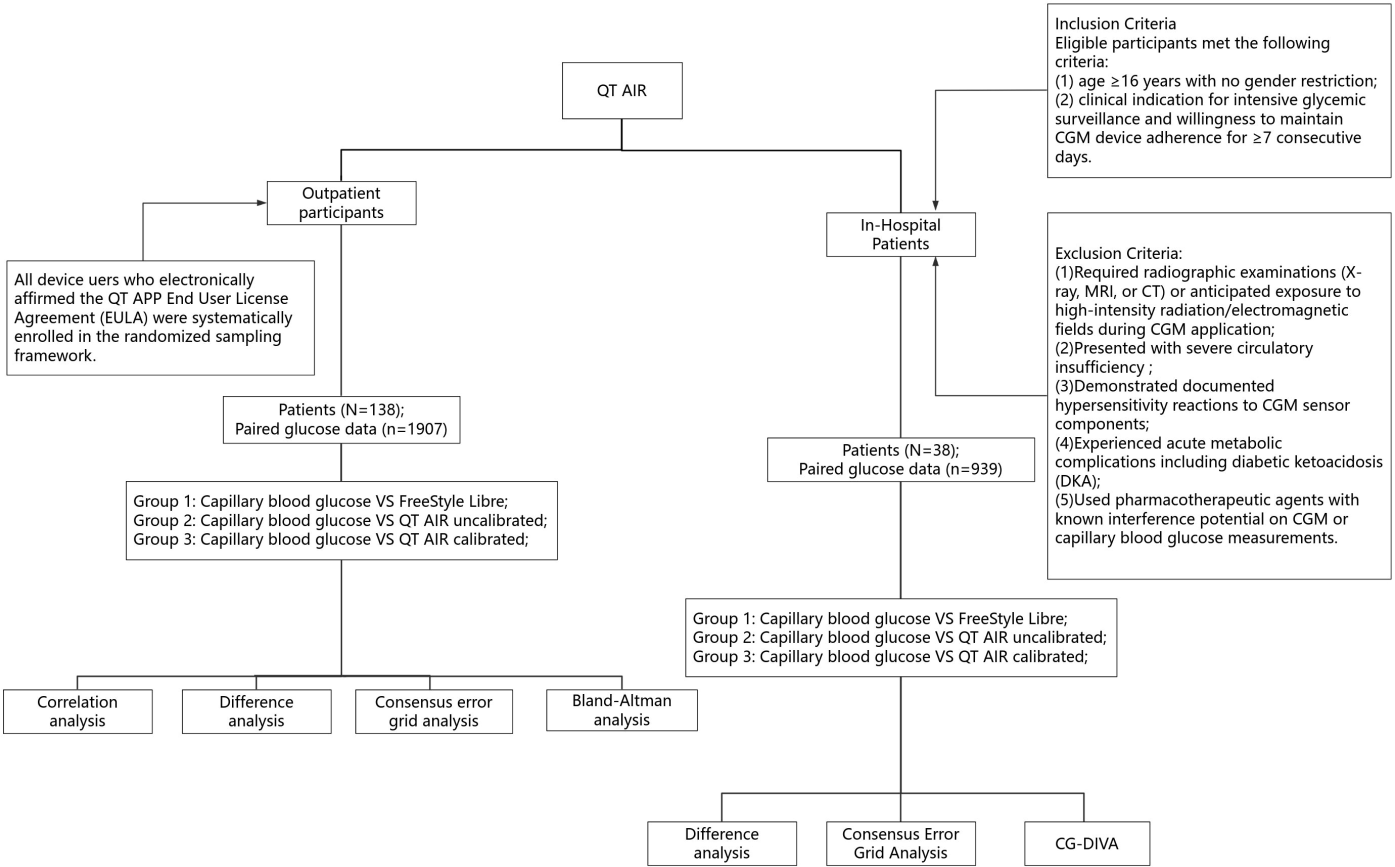
Flowchart of research procedures. CG-DIVA, Glucose Deviation Interval and Variability Analysis.

Statistical analysis was performed using GraphPad Prism 9.0.0 (GraphPad Software, San Diego, CA, USA). The data in accordance with normal distribution were expressed as mean ± standard deviation (). Since the glucose data violated the assumption of normality (assessed by Shapiro-Wilk/Kolmogorov-Smirnov test, p<0.05), Spearman’s rank-order correlation was employed to evaluate the monotonic relationship between variables. Correlation analysis used Bland-Altman analysis (12); p<0.05 was considered as statistically significant. System error analysis used MAD (mean absolute difference) and MARD (mean absolute relative difference) (13); Clinical performance evaluation was conducted by analysis of the consensus error grid (14, 15); Plots and calculations were performed in “Python 3.11.1”. Point accuracy is assessed using Continuous Glucose Deviation Interval and Variability Analysis (CG-DIVA) based on Food and Drug Administration requirements for integrated CGM systems (16) (**Figure 2**).

## 3 Results

### 3.1 The evaluation of the device’s performance in outpatient settings

#### 3.1.1 Correlation analysis

**Figure 3:**
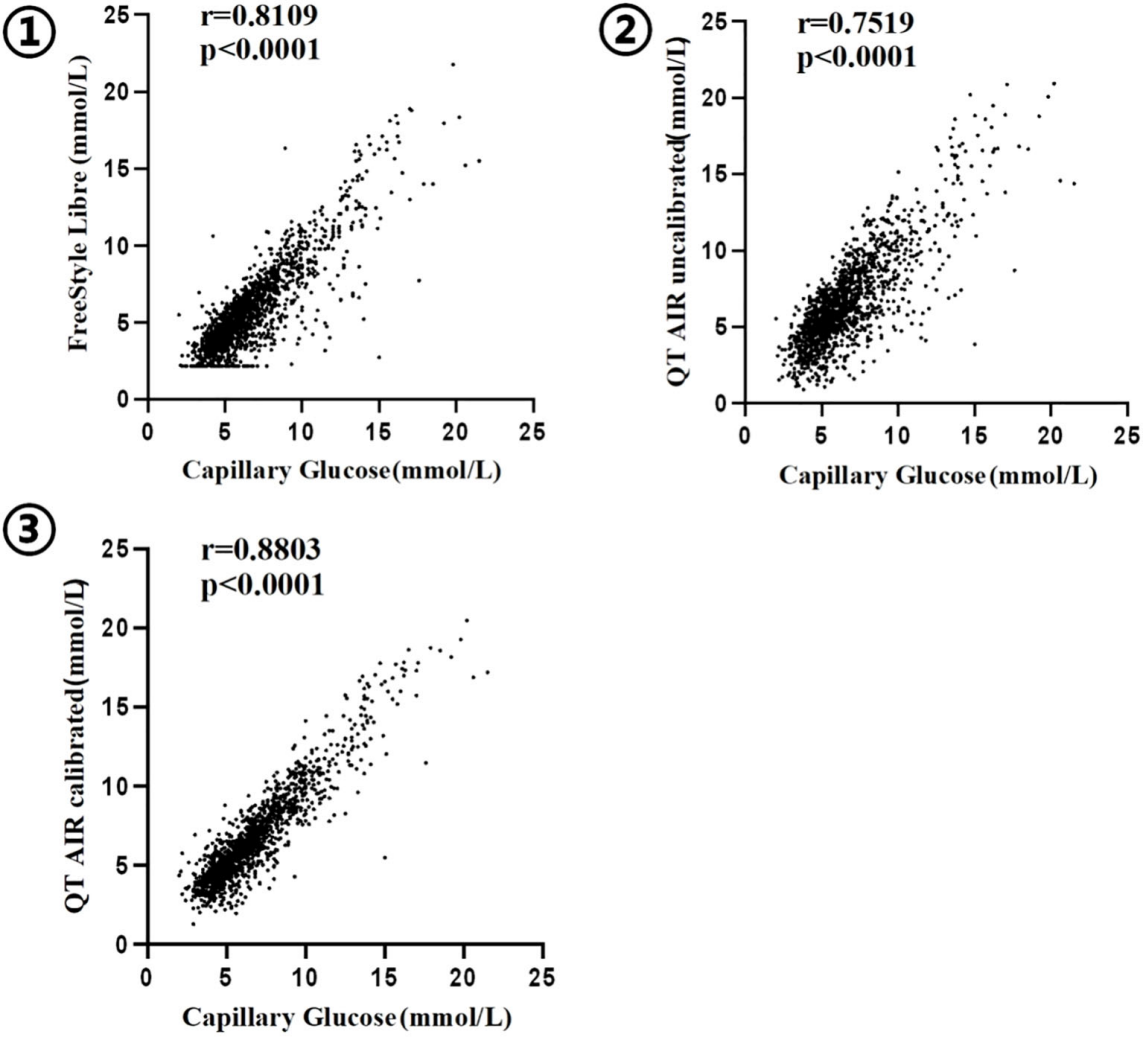
Correlation between capillary blood glucose and CGM ① FreeStyle Libre; ② QT AIR uncalibrated; ③ QT AIR calibrated;.

We have conducted an analysis of 1,907 paired data sets from 138 outpatient users, collected from the QT APP cloud server. The capillary glucose values of the users demonstrated significant and positive Spearman correlation with both Libre data and QT AIR data, before and after calibration (p<0.0001) (Shapiro-Wilk/Kolmogorov-Smirnov test, p<0.001). The correlation ranking of capillary blood glucose levels can be expressed as follows: QT AIR calibrated > Libre > QT AIR uncalibrated (**Figure 3**).

#### 3.1.2 Difference analysis

The MARD for FreeStyle Libre was 18.33% ± 16.31%, while QT AIR uncalibrated has an MARD of 20.63% ± 15.92%. Remarkably, QT AIR calibrated exhibits a significantly superior performance with an MARD of 12.39 ± 12.94%, outperforming the other two groups (overall p < 0.0001).

Further analysis involved stratifying the data based on capillary glucose values.The MAD was used for error comparison in the hypoglycemic group (BG ≤ 3.9 mmol/L,136 samples). The MARD was calculated for the hyperglycemic group (BG > 10.0 mmol/L, 170 samples) and optimal glycemic groups (4.0-10.0 mmol/L, 1601 samples). The results showed that the MAD of QT AIR calibrated data closely approximated that of Libre and significantly outperformed the uncalibrated data in the hypoglycemic group. In the non-hypoglycemic range (BG > 3.9 mmol/L), the MARD values of QT AIR calibrated were significantly better than both the Libre and the uncalibrated group, with Libre slightly surpassing the uncalibrated group (**Table 1**).

**Table 1:** Comparison of MAD and MARD Values Among FreeStyle Libre, Uncalibrated QT AIR, and Calibrated QT AIR in different capillary glucose ranges among Out-of-Hospital Patients.

Capillary glucose range	Sample (n)	FreeStyle Libre	QT AIR uncalibrated	QT AIR calibrated	P value*	P value#
≤3.9mmol/L MAD(mmol/L)	136	0.774 ± 0.6322	0.9204 ± 0.6841	0.7432 ± 0.7311	0.0093	0.7069
4.0-10.0mmol/L MARD(%)	1601	17.97 ± 15.84	20.25 ± 15.08	11.67 ± 10.99	<0.0001	<0.0001
>10.0mmol/L MARD(%)	170	18.13 ± 15.85	18.51 ± 15.17	10.51 ± 9.2	0.6128	<0.0001
>3.9mmol/L MARD(%)	1771	17.98 ± 15.83	20.08 ± 15.1	11.56 ± 10.84	0.0009	<0.0001
Overall MARD(%)	1907	18.33 ± 16.31	20.63 ± 15.92	12.39 ± 12.94	<0.0001	<0.0001

MAD, mean absolute difference. MARD, mean absolute relative difference. *comparison between QT AIR uncalibrated and FreeStyle Libre; #comparison between QT AIR calibrated and FreeStyle Libre.

#### 3.1.3 Consensus error grid analysis

**Figure 4:**
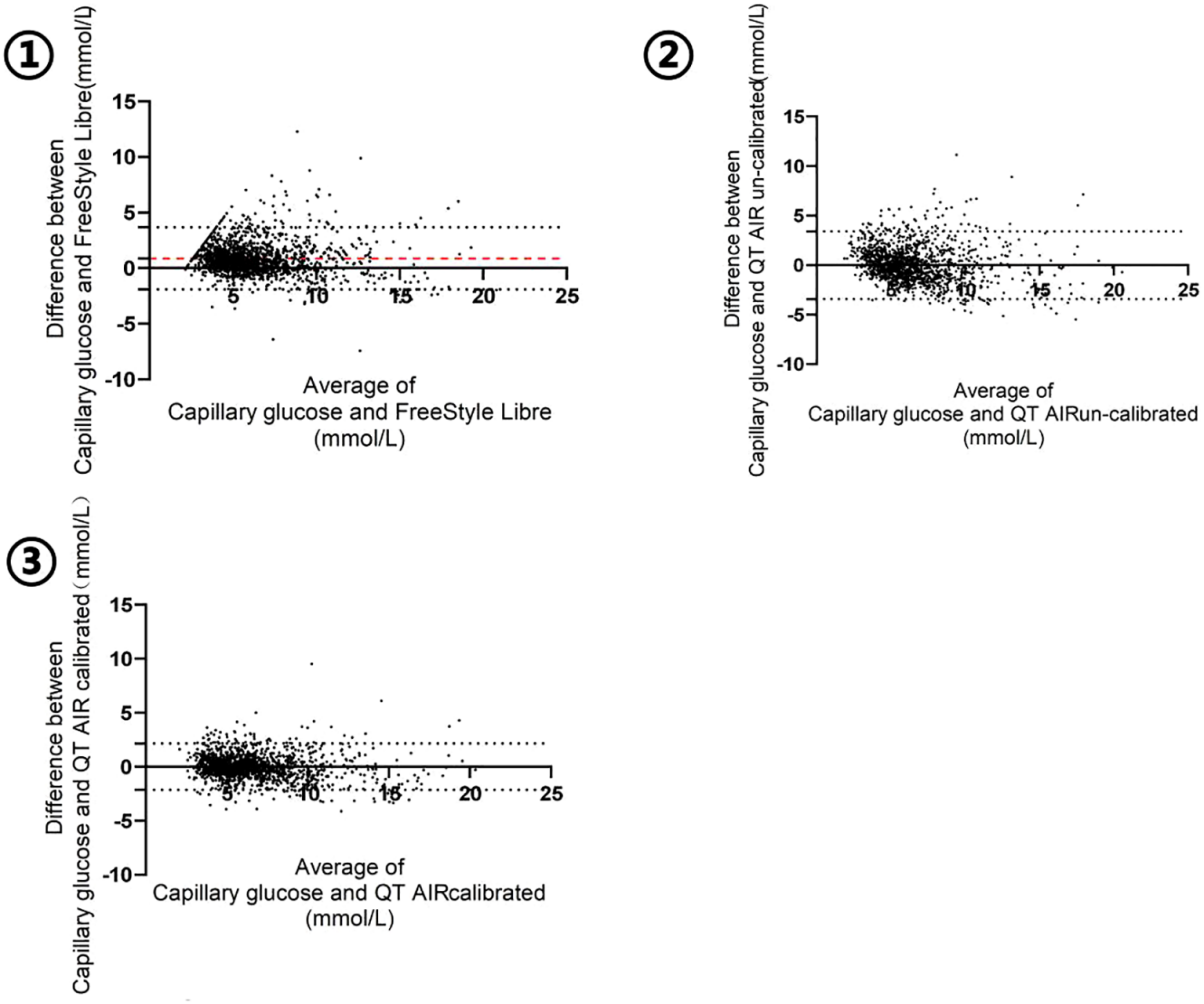
Consensus error grid of Out-of-Hospital Patients. ①comparison between FreeStyle Libre and capillary glucose; ②comparison between QT AIR uncalibrated and capillary glucose; ③comparison between QT AIR calibrated and capillary glucose.

Consensus error grid analysis, an improved version of Clarke error grid analysis, addresses partition incoherence and other limitations to enhance accuracy assessment. The study’s results revealed that the proportions of values falling within regions A and B for FreeStyle Libre, QT AIR calibrated, and QT AIR uncalibrated were all over 99%. However, when focusing on the specific Zone A, the ratio of QT AIR calibrated (87.62%) was significantly superior to that of Libre (69.74%) and QT AIR uncalibrated (67.80%). Remarkably, none of the three exhibited samples in regions D and E, indicating their good performance in avoiding clinically dangerous errors (**Figure 4**, **Table 2**).

**Table 2:** Consensus error grid of out-of-hospital patients.

	FreeStyle Libre		QT AIR uncalibrated		QT AIR calibrated	
	Sample (n)	Percentage (%)	Sample (n)	Percentage (%)	Sample (n)	Percentage (%)
A	1330	69.75	1293	67.80	1671	87.62
B	559	29.31	596	31.26	224	11.75
C	18	0.94	18	0.94	12	0.63
D	0	0.00	0	0.00	0	0.00
E	0	0.00	0	0.00	0	0.00
A+B	1889	99.06	1889	99.06	1895	99.37

#### 3.1.4 Bland-Altman analysis

**Figure 5:**
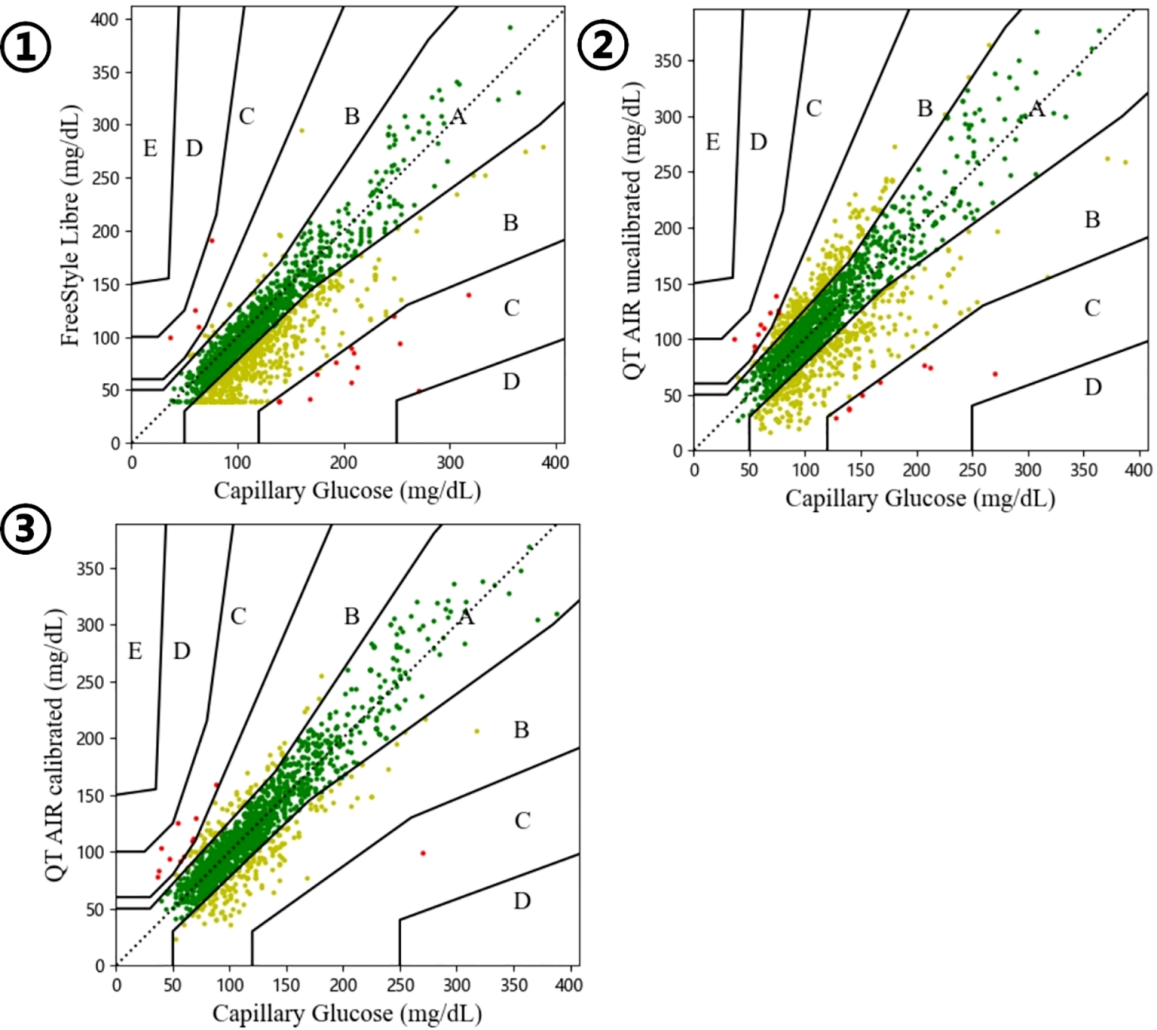
Bland-Altman analysis of FreeStyle Libre, QT AIR calibrated and uncalibrated among Out-of-Hospital Patients.

The Bland-Altman plot was generated with the mean value of capillary glucose and CGM data as the x‐axis and the difference between capillary blood glucose and CGM (BG - CGM) as the y‐axis. As shown in **Figure 5**, the mean difference in FreeStyle Libre was 0.8834 mmol/L, signifying an overall underestimation of FreeStyle Libre compared to capillary glucose data. The mean difference and standard deviation of QT AIR calibrated were significantly lower than those of Libre and QT AIR uncalibrated (**Table 3**).

**Table 3:** Bland-Altman analysis of FreeStyle Libre, QT AIR calibrated and uncalibrated among Out-of-Hospital Patients.

	FreeStyle Libre	QT AIR uncalibrated	QT AIR calibrated
Bias (mmol/L)	0.8834	-0.01517	0.006137
SD of bias	1.423	1.741	1.094
95%CI (mmol/L)	(-1.906,3.673)	(-3.427,3.397)	(-2.137,2.150)

### 3.2 The evaluation of the device’s performance in hospital settings

#### 3.2.1 In-hospital patients characteristics

The clinical characteristics of 38 randomly selected inpatients are as follows: among them, 27 were male and 11 were female, with a mean age of (53.32 ± 17.93) years (range: 18 to 84 years). Among the patients, there were 17 cases of type 1 diabetes, 16 cases of type 2 diabetes, 2 case of reactive hypoglycemia, and 3 cases of latent autoimmune diabetes in adults (LADA). Among these patients, 28 were undergoing insulin therapy, with an average daily insulin dosage of 26.72 ± 22.22 IU. The patients had an average body mass index (BMI) of (24.05 ± 4.62) kg/m² and an average diabetes duration of approximately 11 years (ranging from 1 week to 35 years). The mean value of glycated hemoglobin (HbA1c) was 9.12 ± 2.77% (**Table 4**).

**Table 4:** Characteristics of study subjects.

Variable	Subjects (N=38)
Gender	
Male, N (%)	27 (71.05)
Female, N (%)	11 (28.95)
Diagnosis	
Type 1, N (%)	17 (44.74)
Type 2, N (%)	16 (42.11)
Reactive hypoglycemia, N (%)	2 (5.26)
LADA, N (%)	3 (7.89)
Therapy	
Insulin therapy, N (%)	28 (73.68)
Oral hypoglycemic agents or non-pharmacologically intervened, N (%)	10 (26.32)
Age, mean ± SD, years	53.32 ± 17.93
BMI, mean ± SD, kg/m2	24.05 ± 4.62
HbA1c, %	9.12 ± 2.77

#### 3.2.2 Analysis of blood glucose measurement difference among in-hospital patients

A total of 939 capillary blood glucose data points from in-hospital patients were collected for analysis. The MARD for FreeStyle Libre was calculated as 13.72% ± 14.57%, the uncalibrated QT AIR exhibited a MARD of 13.00% ± 12.44%, and the calibrated QT AIR yielded a significantly improved MARD of 7.27% ± 7.45%. The statistical analysis showed a significant difference among these MARD values (p<0.0001).

#### 3.2.3 Consensus error grid analysis of blood glucose among in-hospital patients

**Figure 6:**
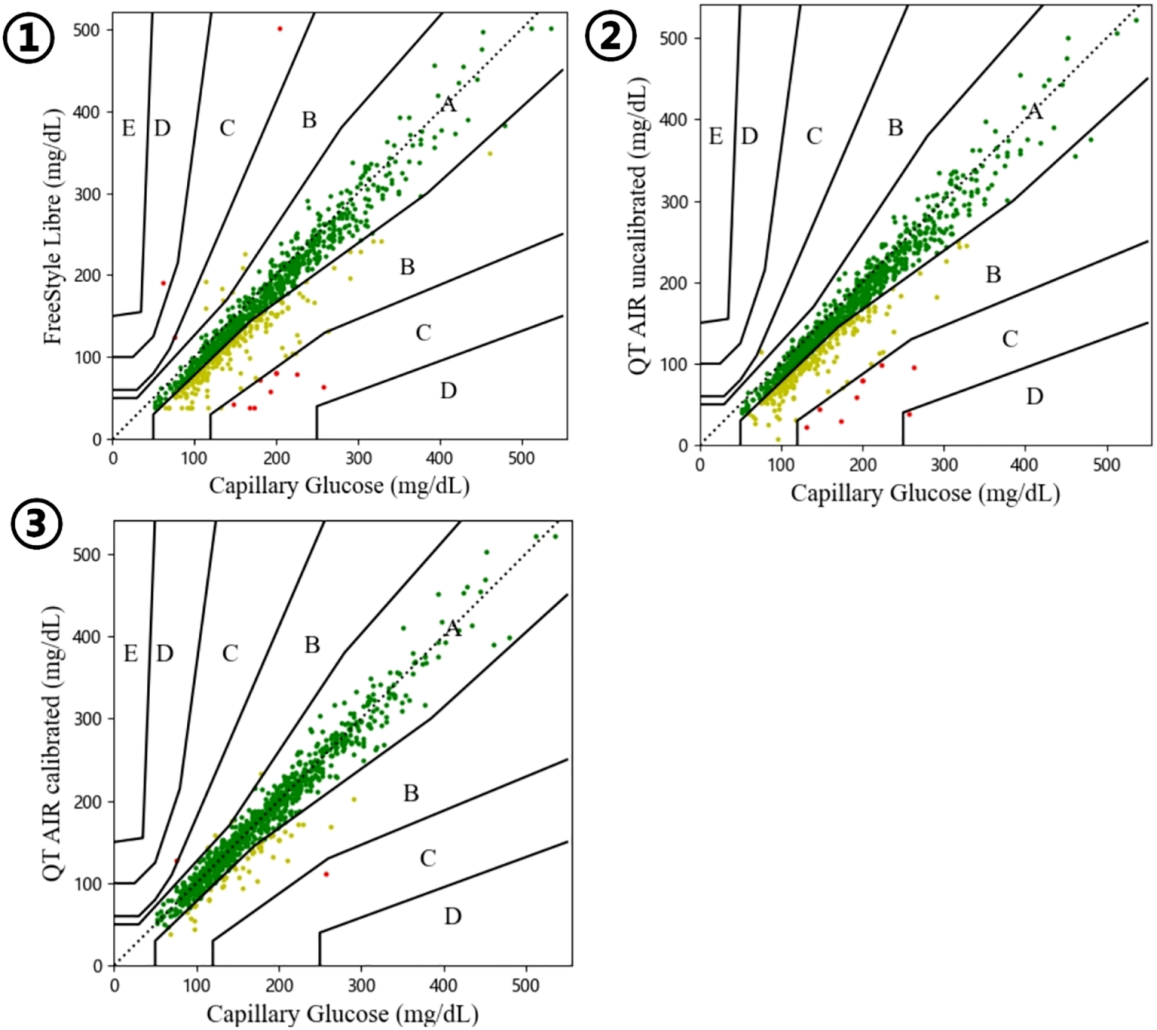
Consensus error grid among In-Hospital Patients. ①comparison between FreeStyle Libre and capillary glucose; ②comparison between QT AIR uncalibrated and capillary glucose; ③comparison between QT AIR calibrated and capillary glucose.

As illustrated in **Figure 6**, **Table 5**, the consensus error grid analysis for in-hospital patients revealed that approximately 99% of the data points from Libre, uncalibrated QT AIR, and calibrated QT AIR fell within the A+B zones. Similar to the outpatient results, the calibrated QT AIR exhibited a substantial improvement with 94.89% of its data points falling within the A zone, surpassing the 78.70% of Libre and the 79.45% of uncalibrated QT AIR.

**Table 5:** Consensus error grid among In-hospital patients.

Zone	FreeStyle Libre		QT AIR uncalibrated		QT AIR calibrated	
	Sample (n)	Percentage (%)	Sample (n)	Percentage (%)	Sample (n)	Percentage (%)
A	739	78.70	746	79.45	891	94.89
B	188	20.02	184	19.59	46	4.90
C	11	1.17	8	0.85	2	0.21
D	1	0.11	1	0.11	0	0.00
E	0	0.00	0	0.00	0	0.00
A+B	927	98.72	930	99.04	937	99.79

#### 3.2.4 Point accuracy

**Figure 7:**
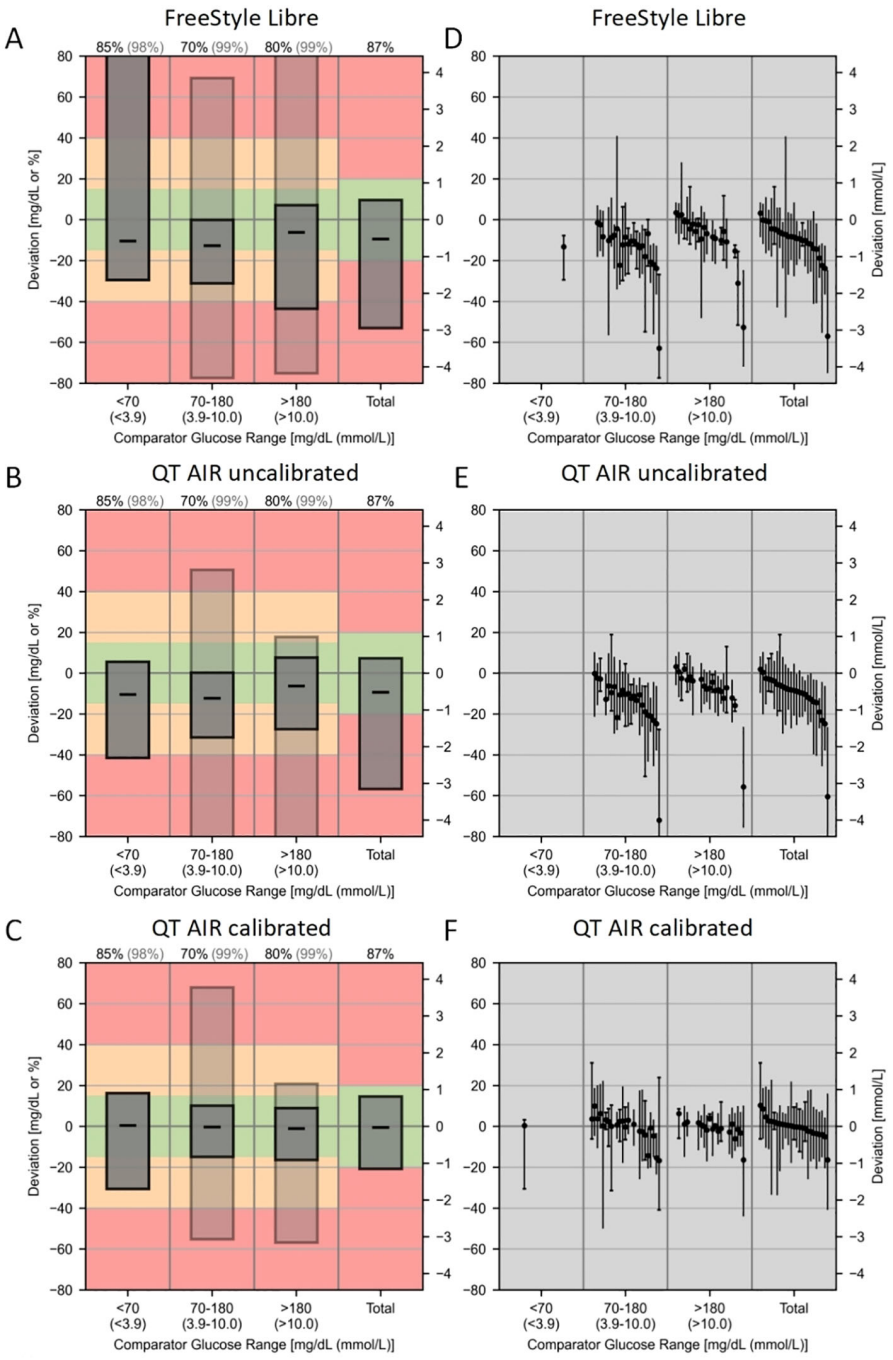
Continuous Glucose Deviation Interval and Variability Analysis (CG-DIVA) of FreeStyle Libre, QT AIR uncalibrated, and calibrated. **(A–C)**: Provides an absolute bias comparison for glucose levels <70 mg/dL and a relative bias comparison for all other glucose levels, referencing the right axis scale. Consistency rates (AR) are used to evaluate the other glucose ranges, referencing the left axis scale.The bias intervals are shown as light/dark gray boxes, indicating the expected range of biases. The median bias is represented by a black dashed line. The coverage of the bias intervals, i.e., the percentage of biases falling within the intervals, is set according to the requirements defined by the U.S. Food and Drug Administration (FDA) and is printed at the top of the figure.The dark gray box represents standard I, and the light gray box represents standard II, corresponding to the percentage colors of the bias ranges at the top.The colored background indicates the degree of bias and is based on the limits of standards I and II. The green range represents standard I, the yellow range, which includes the green, represents standard II, and the red indicates exceeding the standard requirements. **(D–F)**: Each sensor is described by the range of bias within the median and the 90% range, and sensors are ranked by median bias within the overall glucose range, with black solid dots representing the median positions.

The CG-DIVA results are shown in the **Figure 7**. **Figures 7A–C**, show the overall distribution of CGM reading biases across different glucose ranges. Overall median biases were -9.40%, -9.36%, and -0.49% for Libre, QT AIR uncalibrated, and calibrated, respectively. **Figures 7D–F** shows the variability of different representative sensors across various glucose levels, with an overall between sensor variability range of 60.17%, 62.40%, and 26.65% for Libre, QT AIR uncalibrated, and calibrated, respectively.

## 4 Discussion

The CGM detects blood glucose through a series of interstitial fluid glucose measurement. It is very helpful for diabetic patients to optimize their treatment regimens (17). CGM systems are categorized into retrospective and real-time dynamic glucose monitoring systems. Real-time CGM devices provide a real-time glucose reading and glucose change trend. It not only facilitates convenient glucose information retrieval but also enables the anticipation of hyperglycemic or hypoglycemic events. As a result, users are empowered to promptly adjust their glycemic management strategies, thereby increase their TIR and improving overall glycemic control (18). However, FreeStyle Libre, a widely used intermittently scanned CGMs in China and other developing countries, operates as a retrospective system. Based on this technical foundation, our research team developed QT AIR to address these limitations. QT AIR upgraded Libre to a calibratable real-time continuous blood glucose monitoring device.

Capillary blood glucose, obtained from fingertip capillaries, remains an irreplaceable clinical prominence as the most commonly employed glycemic reference metric in both patients’ daily lives and clinical blood glucose management practices (19). Considering its reliablility and convienence, we utilize CBG as the benchmark for comparing the values obtained from CGM systems.

The evaluation of the accuracy of CGM systems encompasses two aspects: numerical accuracy and clinical accuracy assessment. In terms of numerical accuracy, the consistency analysis results reveal significant correlations among all three groups of outpatient users—Libre, calibrated QT AIR, and uncalibrated QT AIR. Among these, calibrated QT AIR exhibits the highest degree of correlation. The Bland-Altman analysis serves as a quantitative and directional means of assessing the consistency between two different measurement methods. In the comparison between FreeStyle Libre readings and capillary blood glucose values, it is evident that the Libre generally underestimates the blood glucose. This observation aligns with conclusions drawn from other relevant studies (10, 11). Comparatively, the differences between QT AIR monitoring values and capillary blood glucose values are less pronounced, with the average difference narrowing further after calibration. MARD is the measure of choice for evaluating the accuracy of CGM systems. Calibrated QT AIR exhibits a significant reduction in MARD values when compared to both Libre and uncalibrated QT AIR. In the outpatient setting, among the FreeStyle Libre, uncalibrated QT AIR and calibrated QT AIR, Consensus error grid analyses demonstrated data distribution exceeding 97% in zones A+B. Furthermore, after the calibration of QT AIR, the data within zone A rising from less than 70% to over 80%, resulting in a noteworthy enhancement in clinical accuracy within the context of daily outpatient scenarios. Building upon the factory calibration of FreeStyle Libre, QT AIR introduces capillary blood calibration to further refine data accuracy, thereby facilitating superior monitoring outcomes for users.

In the context of outpatient CGM device utilization, inaccuracies in measurement data may stem from various factors, including improper self-application of the sensor, intricate physical activities leading to suboptimal sensor adherence, and other related issues. Moreover, the accuracy of capillary blood glucose values, utilized as a calibration reference, could be influenced by variances in the patient’s self-sampling techniques, inconsistent skin disinfection procedures, and variations in the quality of testing instruments (20, 21). Therefore, our data collection was conducted among inpatients as well, with the assistance of specialized nursing staff, to ensure proper device placement, conduct regular checks of device functionality, and implement standardized procedures for capillary blood sampling. These measures were undertaken to mitigate potential data inaccuracies arising from procedural variations. In the hospital setting, the MARD of FreeStyle Libre was 13.72%, which closely approximates the official claim of 11.4%. However, after rigorous capillary blood calibration, the MARD of QT AIR decreased significantly to 7.27%, surpassing FreeStyle Libre with factory calibration. Additionally, this MARD value was lower than the reported 9.2% for subsequent iterations of FreeStyle Libre (22, 23). Previous studies have demonstrated that the MARD values of Dexcom G7 were 8.2% or 9.1%, compared with 9.4% for Guardian™ Sensor 3 (24, 25). After calibration, QT AIR exhibited comparable performance to these market-leading glucose monitoring systems. In terms of clinical accuracy, similar to the results of outpatients, the Consensus error grid analysis revealed that the predominant data points for all three groups fell within zones A and B. However, after calibration, QT AIR improved the proportion of data points in zone A from approximately 80% to 94.89%. This outcome underscores the remarkable clinical accuracy of QT AIR, underscoring its potential to deliver monitoring outcomes akin to capillary blood glucose levels when subjected to meticulous adherence to wearing protocols and rigorous data calibration procedures. CG-DIVA results show that after algorithm optimization and calibration, the QT AIR significantly improved the point accuracy of Libre and reduced the variability between devices and within devices. Furthermore, the calibrated QT AIR’s point accuracy has approached the benchmark required for an Integrated-CGM system.

Our study has certain limitations. In the outpatient study, there is a potential occurrence of inadequate sensor adherence. However, we addressed this concern by implementing data collection within a separated population, well monitored inpatients, and got a consistent result. The sample size in the hospital data collection was limited. We did not incorporate a gold standard reference such as venous blood for comparison.

## 5 Conclusion

In summary, we have successfully upgraded the intermittently scannedCGM device to a real-time CGM device and introduced capillary blood calibration to enhance accuracy. From the results, QT AIR demonstrated statistically significant enhancements in both data accuracy and clinical accuracy after calibration, surpassing the performance of FreeStyle Libre and achieving parity with leading CGM devices.

## Data Availability

The raw data supporting the conclusions of this article will be made available by the authors, without undue reservation.

## References

[1] Standards of medical care in diabetes-2022 abridged for primary care providers. Clin Diabetes. (2022) 40:10–38. doi: 10.2337/cd22-as01 35221470 PMC8865785

[2] Lovic D Piperidou A Zografou I Grassos H Pittaras A Manolis A. The growing epidemic of diabetes mellitus. Curr Vasc Pharmacol. (2020) 18:104–9. doi: 10.2174/1570161117666190405165911 30961501

[3] The Prevention of Diabetes Mellitus. JAMA. (2021) 325(2):190. doi: 10.1001/jama.2020.17738 33433568

[4] Galindo RJ Aleppo G. Continuous glucose monitoring: the achievement of 100 years of innovation in diabetes technology. Diabetes Res Clin Pract. (2020) 170:108502. doi: 10.1016/j.diabres.2020.108502 33065179 PMC7736459

[5] Bolli GB. Hypoglycaemia unawareness. Diabetes Metab. (1997) 23 Suppl 3:29–35. doi: 10.5114/pedm.2018.80994 9342540

[6] Battelino T Danne T Bergenstal RM Amiel SA Beck R Biester T. Clinical targets for continuous glucose monitoring data interpretation: recommendations from the international consensus on time in range. Diabetes Care. (2019) 42:1593–603. doi: 10.2337/dci19-0028 PMC6973648 31177185

[7] Blum A. Freestyle libre glucose monitoring system. Clin Diabetes. (2018) 36:203–4. doi: 10.2337/cd17-0130 PMC5898159 29686463

[8] Freckmann G Pleus S Grady M Setford S Levy B. Measures of accuracy for continuous glucose monitoring and blood glucose monitoring devices. J Diabetes Sci Technol. (2019) 13:575–83. doi: 10.1177/1932296818812062 PMC6501529 30453761

[9] Edelman SV Argento NB Pettus J Hirsch IB. Clinical implications of real-time and intermittently scanned continuous glucose monitoring. Diabetes Care. (2018) 41:2265–74. doi: 10.2337/dc18-1150 30348844

[10] Zhang Y Liu X Zhang J Fu J Li S Chen S. Evaluation for the feasibility and accuracy of freestyle libre flash glucose monitoring system used by covid-19 patients in intensive care unit. J Diabetes. (2021) 13:603–5. doi: 10.1111/1753-0407.13181 PMC8250832 33787006

[11] Aberer F Hajnsek M Rumpler M Zenz S Baumann PM Elsayed H. Evaluation of subcutaneous glucose monitoring systems under routine environmental conditions in patients with type 1 diabetes. Diabetes Obes Metab. (2017) 19:1051–5. doi: 10.1111/dom.12907 28205324

[12] Bland JM Altman DG. Statistical methods for assessing agreement between two methods of clinical measurement. Lancet. (1986) 1:307–10. doi: 10.1016/S0140-6736(86)90837-8 2868172

[13] Reiterer F Polterauer P Schoemaker M Schmelzeisen-Redecker G Freckmann G Heinemann L. Significance and reliability of mard for the accuracy of cgm systems. J Diabetes Sci Technol. (2017) 11:59–67. doi: 10.1177/1932296816662047 27566735 PMC5375072

[14] Clarke WL Cox D Gonder-Frederick LA Carter W Pohl SL. Evaluating clinical accuracy of systems for self-monitoring of blood glucose. Diabetes Care. (1987) 10:622–8. doi: 10.2337/diacare.10.5.622 3677983

[15] Parkes JL Slatin SL Pardo S Ginsberg BH. A new consensus error grid to evaluate the clinical significance of inaccuracies in the measurement of blood glucose. Diabetes Care. (2000) 23:1143–8. doi: 10.2337/diacare.23.8.1143 10937512

[16] Eichenlaub M Stephan P Waldenmaier D Pleus S Rothenbühler M Haug C. Continuous glucose deviation interval and variability analysis (Cg-diva): A novel approach for the statistical accuracy assessment of continuous glucose monitoring systems. J Diabetes Sci Technol. (2024) 18(4)::857–65. doi: 10.1177/19322968221134639 PMC11307236 36329636

[17] Rodbard D. Continuous glucose monitoring: A review of recent studies demonstrating improved glycemic outcomes. Diabetes Technol Ther. (2017) 19:S25–s37. doi: 10.1089/dia.2017.0035 28585879 PMC5467105

[18] Préau Y Galie S Schaepelynck P Armand M Raccah D. Benefits of a switch from intermittently scanned continuous glucose monitoring (Iscgm) to real-time (Rt) cgm in diabetes type 1 suboptimal controlled patients in real-life: A one-year prospective study (§). Sensors (Basel). (2021) 21:6131. doi: 10.3390/s21186131 34577338 PMC8473395

[19] Heinemann L Freckmann G Koschinsky T. Considerations for an institution for evaluation of diabetes technology devices to improve their quality in the European Union. J Diabetes Sci Technol. (2013) 7:542–7. doi: 10.1177/193229681300700230 PMC3737655 23567012

[20] Ginsberg BH. Factors affecting blood glucose monitoring: sources of errors in measurement. J Diabetes Sci Technol. (2009) 3:903–13. doi: 10.1177/193229680900300438 PMC2769960 20144340

[21] Freckmann G Jendrike N Baumstark A Pleus S Liebing C Haug C. User performance evaluation of four blood glucose monitoring systems applying ISO 15197:2013 accuracy criteria and calculation of insulin dosing errors. Diabetes Ther. (2018) 9:683–97. doi: 10.1007/s13300-018-0392-6 PMC6104257 29502304

[22] Alva S Bailey T Brazg R Budiman ES Castorino K Christiansen MP. Accuracy of a 14-day factory-calibrated continuous glucose monitoring system with advanced algorithm in pediatric and adult population with diabetes. J Diabetes Sci Technol. (2022) 16:70–7. doi: 10.1177/1932296820958754 PMC8875061 32954812

[23] Bailey T Bode BW Christiansen MP Klaff LJ Alva S. The performance and usability of a factory-calibrated flash glucose monitoring system. Diabetes Technol Ther. (2015) 17:787–94. doi: 10.1089/dia.2014.0378 PMC4649725 26171659

[24] Garg SK Kipnes M Castorino K Bailey TS Akturk HK Welsh JB. Accuracy and safety of dexcom G7 continuous glucose monitoring in adults with diabetes. Diabetes Technol Ther. (2022) 24:373–80. doi: 10.1089/dia.2022.0011 PMC9208857 35157505

[25] Christiansen MP Garg SK Brazg R Bode BW Bailey TS Slover RH. Accuracy of a fourth-generation subcutaneous continuous glucose sensor. Diabetes Technol Ther. (2017) 19:446–56. doi: 10.1089/dia.2017.0087 PMC5567873 28700272

