# Supplementary material for: Accuracy of a novel calibratable real-time continuous glucose monitoring device based on FreeStyle libre in- and out-of-hospital
Source: Front Endocrinol (Lausanne). 2025 Apr 22;16:1466358. doi: 10.3389/fendo.2025.1466358 (PMC12053481; doi:10.3389/fendo.2025.1466358)
Supplement: Supplementary file 1 [file Table1.docx]

Supplementary Material

| Glucose range | Median bias | | | Standard I | | | Standard II | | |
| --- | --- | --- | --- | --- | --- | --- | --- | --- | --- |
|  | FreeStyle Libre | QT AIR uncalibrated | QT AIR calibrated | FreeStyle Libre | QT AIR uncalibrated | QT AIR calibrated | FreeStyle Libre | QT AIR uncalibrated | QT AIR calibrated |
| <70mg/dL  (mg/dL) | -10.41 | -10.46 | 0.45 | [-29.43 , 128.94] | [-41.47 , 5.54] | [-30.61 , 16.19] | [-29.43 , 128.94 ] | [-41.47 , 5.54 ] | [-30.61 , 16.19] |
| 70-180mg/dL(%) | -12.70 | -12.24 | -0.29 | [-31.12 , -0.03] | [-31.46 , 0.25] | [-14.93 , 10.20] | [-77.43 , 69.31 ] | [-92.67 , 50.64] | [-55.14 , 67.93] |
| >180mg/dL(%) | -6.21 | -6.25 | -1.01 | [-43.56 , 7.08] | [-27.42 , 7.69] | [-16.42 , 8.97] | [-75.14 , 146.31] | [-85.25 , 17.73] | [-56.82 , 20.87] |
| Total(%) | -9.40 | -9.37 | -0.49 | [-52.96, 9.64] | [-56.71 , 7.27 ] | [-20.81 , 14.67 ] | - | - | - |

Table 1 Continuous Glucose Deviation Interval of FreeStyle Libre, QT AIR uncalibrated, and calibrated.
